# Identification of G8969>A in mitochondrial *ATP6* gene that severely compromises ATP synthase function in a patient with IgA nephropathy

**DOI:** 10.1038/srep36313

**Published:** 2016-11-04

**Authors:** Shuzhen Wen, Katarzyna Niedzwiecka, Weiwei Zhao, Shutian Xu, Shaoshan Liang, Xiaodong Zhu, Honglang Xie, Déborah Tribouillard-Tanvier, Marie-France Giraud, Caihong Zeng, Alain Dautant, Róża Kucharczyk, Zhihong Liu, Jean-Paul di Rago, Huimei Chen

**Affiliations:** 1 National Clinical Research Center of Kidney Diseases, Jinling Hospital, Nanjing University School of Medicine, Nanjing, China; 2 Institute of Biochemistry and Biophysics, Polish Academy of Sciences, Warsaw, Poland; 3 CNRS, Institut de Biochimie et Génétique Cellulaires, UMR 5095, F-33077 Bordeaux, France; 4 Université de Bordeaux, IBGC, UMR 5095, F-33077 Bordeaux, France; 5 INSERM, Institut de Biochimie et Génétique Cellulaires, F-33077 Bordeaux, France.

## Abstract

Here we elucidated the pathogenesis of a 14-year-old Chinese female who initially developed an isolated nephropathy followed by a complex clinical presentation with brain and muscle problems, which indicated that the disease process was possibly due to a mitochondrial dysfunction. Careful evaluation of renal biopsy samples revealed a decreased staining of cells induced by COX and NADH dehydrogenase activities, and a strong fragmentation of the mitochondrial network. These anomalies were due to the presence of a mutation in the mitochondrial *ATP6* gene, G8969>A. This mutation leads to replacement of a highly conserved serine residue at position 148 of the *a*-subunit of ATP synthase. Increasing the mutation load in cybrid cell lines was paralleled by the appearance of abnormal mitochondrial morphologies, diminished respiration and enhanced production of reactive oxygen species. An equivalent of the G8969>A mutation in yeast had dramatic consequences on ATP synthase, with a block in proton translocation. The mutation was particularly abundant (89%) in the kidney compared to blood and urine, which is likely the reason why this organ was affected first. Based on these findings, we suggest that nephrologists should pay more attention to the possibility of a mitochondrial dysfunction when evaluating patients suffering from kidney problems.

Mitochondrial dysfunction has been implicated in a broad spectrum of human diseases, often referred to as mitochondrial cytopathies (MCs). These diseases affect at least 1 in 5,000 live human births^1^, and can present either in infancy or adulthood, in a multi-systemic or highly tissue-specific manner. Typical clinical traits include visual/hearing defects, encephalopathies, cardiomyopathies, myopathies, diabetes, liver and renal dysfunctions^2^,^3^. Most of the known (>150) mitochondrial dysfunction genetic syndromes that have been described arise from disorders affecting oxidative phosphorylation, which is a mitochondrial process that provides cellular energy by generating ATP molecules.

The energy-transducing system of mitochondria comprises multi-subunit complexes (I-V) embedded within the mitochondrial inner membrane, which together with cytochrome *c* and ubiquinone, form what is usually called the OXPHOS system^4^. CI-IV transfer electrons to oxygen, a process that is coupled to the pumping of protons out of the mitochondrial matrix; protons are transported back into the matrix by CV (ATP synthase), which is coupled to ATP synthesis from ADP and inorganic phosphate. The OXPHOS system has a mixed genetic origin, nuclear and mitochondrial. It contains approximately 90 different structural protein subunits of which thirteen are encoded by the mtDNA in humans^5^.

We here report a Chinese girl who was admitted to hospital at the age of 14 for a severe IgA nephropathy. This is a common glomerulonephritis typically affecting young adults that can also occur in children and the elderly. The disease has a wide spectrum of clinical symptoms, ranging from asymptomatic microscopic hematuria to a more severe course characterized by sustained proteinuria and rapid deterioration of renal function^6^. Unexpectedly, our patient further developed a more complex clinical presentation with brain and muscle problems, indicating that the disease had possibly a mitochondrial origin. We indeed found that a mutation in the mtDNA that dramatically compromises mitochondrial ATP production contributed to the disease process.

## Results

### Case report

**Figure 1:**
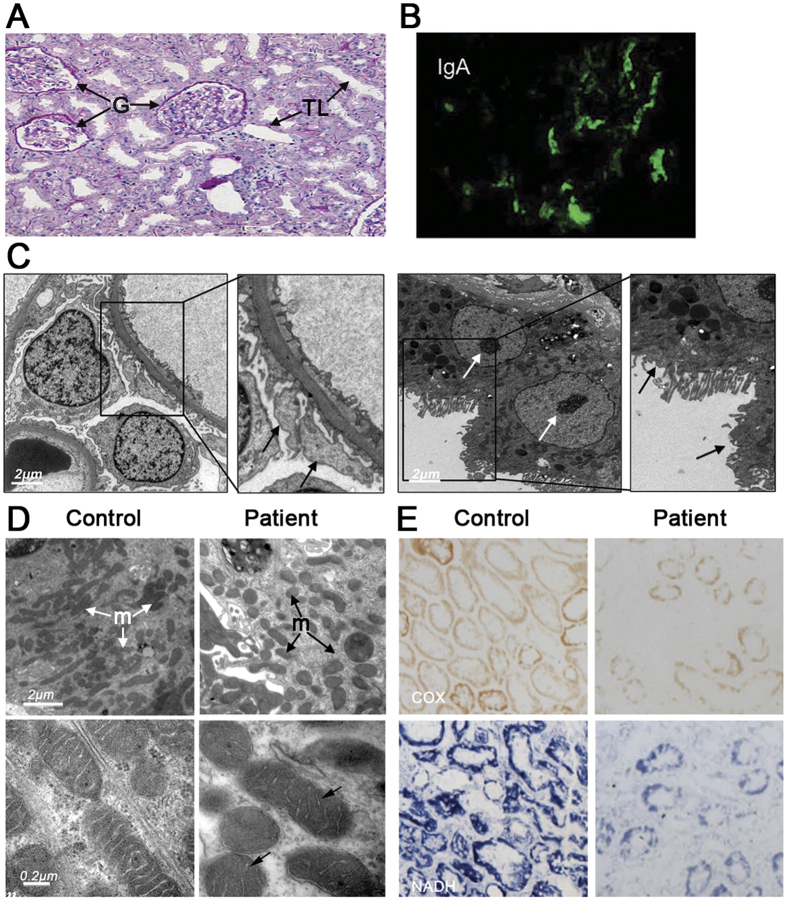
Kidney defects in the patient. (**A**) Light microscopy of kidney cells shows minimal change disease (MCD) in glomeruli (G) and tubular lumen (TL) dilatation in the patient. (**B**) Fluorescence microscopy shows IgA deposits in kidney cells (*vs* black balanced control cells). (**C**) Electron micrographs showing podocyte foot process fusion in glomeruli (left, ↑), electron-dense deposits in the glomerular mesangium (white arrows) and brush border loss in focal tubules (right, ↑). (**D**) Ultrastructure of mitochondria (m) in tubular epithelial cells from the patient and a control individual. (**E**) *In situ* cytochrome *c* oxidase (COX) and NADH dehydrogenase activities in renal tissue from the patient and a control individual.

The patient was admitted at the age of 14 to the hospital due to a nephrotic syndrome characterized by the presence of high levels of red blood cells and protein in urine, and edema. A complete remission was observed after treatment with steroids, but she relapsed two years later. Following readmission, a renal biopsy was taken and shown to have almost normal glomeruli with only mild mesangial widening, whereas segmental tubular epithelial cells were flat and the interstitium between tubules were abnormally enlarged (Fig. 1A). Immunofluorescence analyses revealed the presence of immunoglobulin A (IgA) deposits in the kidney, a typical sign of IgA nephropathy (Fig. 1B)^6^. Further evidence for kidney alteration was provided by electron microscopy, which revealed podocyte foot process fusion in glomeruli, the presence of electron-dense deposits in the glomerular mesangium, and brush border loss in focal tubules (Fig. 1C, see ref. 7 for electron micrographs of healthy kidney tissues).

The patient was treated again with steroids and entered remission for a second time. However, two years later renal impairment reoccurred and the patient was hospitalized once more for serious edema and very low urine output (oliguria). Meanwhile, she showed epileptic episodes and decreased muscle strength, brain atrophy, severe hearing impairment, Wolff-Parkinson-White syndrome, and increased fasting level of glucose (11.09 mM *vs* 3.6 mM for the control).

### Hints for mitochondrial dysfunction

Mitochondria in tubular epithelial cells of the patient had a rather spherical contour whereas they were much more elongated in control cells (Fig. 1D), indicating an enhanced fragmentation of the mitochondrial network. Histochemical analyses of fresh renal biopsies further revealed that cytochrome *c* oxidase (COX) and nicotinamide adenine dinucleotide (NADH) dehydrogenase activities were substantially decreased in the patient compared to the control (Fig. 1E, Supplementary Fig. S1). We additionally detected a pronounced peak of lactate in cerebrospinal fluid from the patient (Supplementary Fig. S2) which indicated a less efficient oxidation of pyruvate (the oxidized form of lactate) in mitochondria.

**Figure 2:**
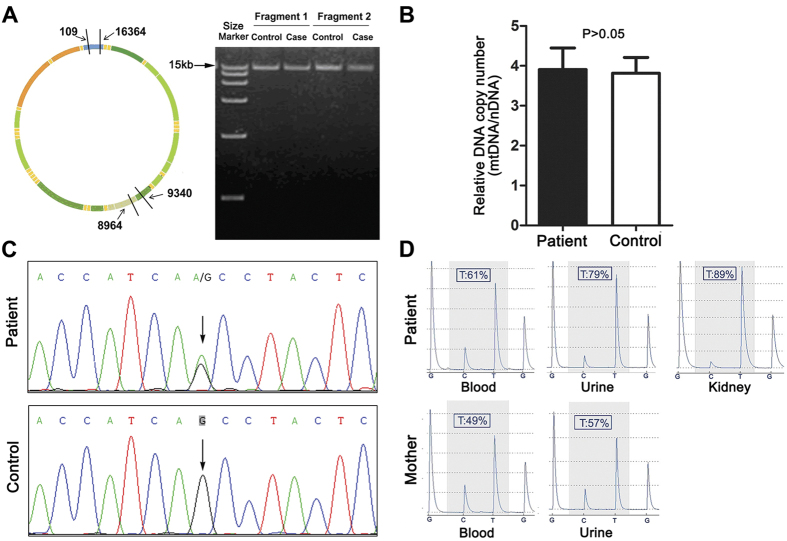
Analyses of the mtDNA of the patient. (**A**) The mtDNAs from the patient and control cells were investigated by PCR using two primers from positions 109 to 16364 (16255bp, fragment 1), and 9340 to 8964 (16250bp, fragment 2), as depicted on the left, and agarose gel analysis of the amplified DNA fragments (right), which revealed the absence of deletion in the mtDNA of the patient. (**B**) Relative mtDNA copy number in the patient and control as measured by real-time quantitative PCR. (**C**) Partial mtDNA sequence chromatogram showing the presence at position 8969 in the patient of both the wild type (Guanine (G), in black) and mutant (Adenine (A), in green) nucleotides. (**D**) Percentages of the G8969>A mutation in the blood and urine sediments of the patient’s mother, and in urine sediments, blood and kidney of the patient, as determined by pyrosequencing.

These observations suggested that a mitochondrial dysfunction was possibly at the origin of, or contributed to, the disease process. This led us to analyze the mtDNA of the patient. No visible rearrangement (deletion) (Fig. 2A) and depletion (Fig. 2B) were observed. However, a point mutation (G8969>A) that was absent in 2704 controls in databases as well as in 100 age-matched controls from the same geographic region, was detected in the *ATP6* gene (Fig. 2C, Supplementary Table S3). This mutation leads to the replacement of a serine residue by asparagine at position 148 in the *a*-subunit of ATP synthase (*a*S148). This residue strictly conserved in mitochondria and bacteria (see below).

Pyrosequencing analysis revealed that the G8969>A mutation was heteroplasmic in blood, urine sediments (epithelial-like cells detached from tubules) and kidney, with loads ranging from 60 to 90% respectively (Fig. 2D). The G8969>A mutation was also detected in blood and urine sediments from the mother, indicating that the mutation was maternally inherited. It was however in lower amounts in the mother (<60%, Fig. 2D), which may explain that she was free of symptoms. It is indeed well known that mtDNA mutations become deleterious for health only beyond a certain threshold^8^, usually above 70% in the case of *ATP6* mutations^9^.

### Properties of cybrid cell lines containing various proportions of the G8969>A mutation

**Figure 3:**
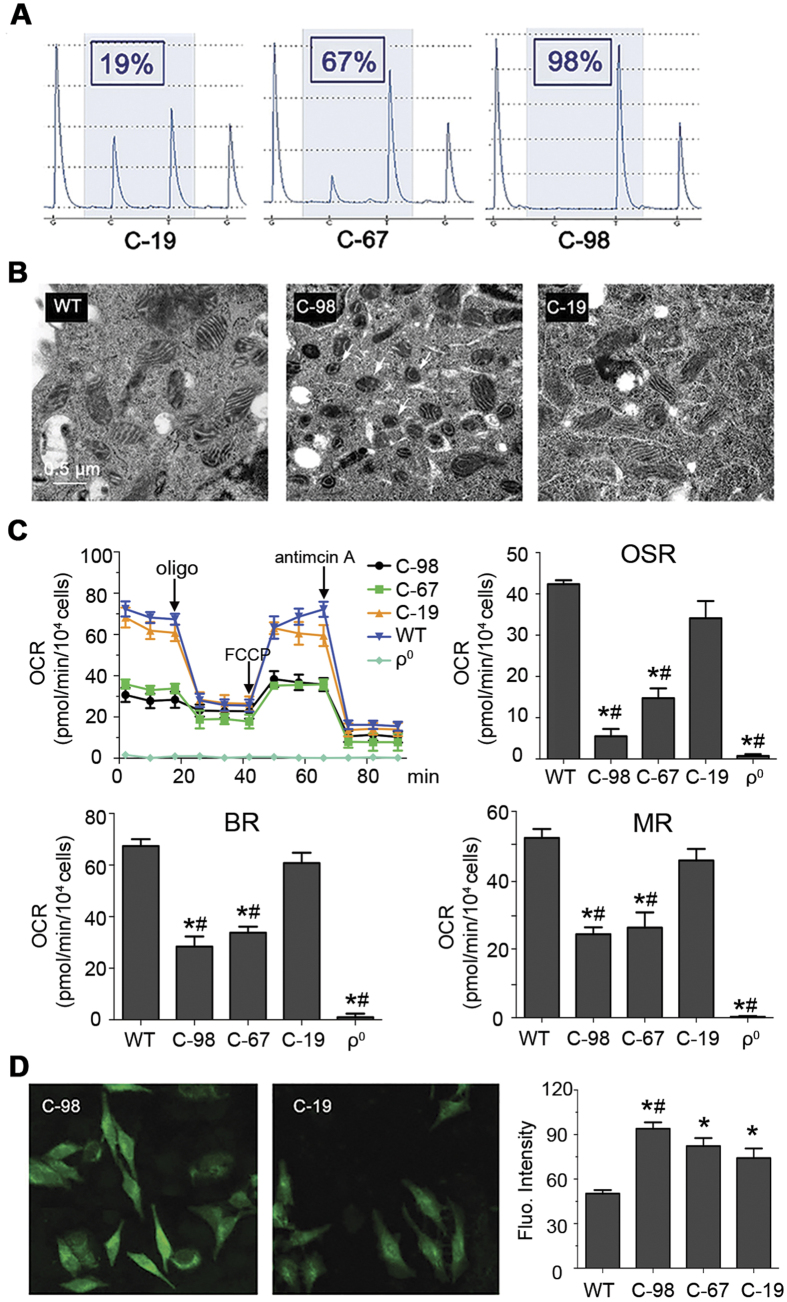
Properties of trans-mitochondrial cell lines carrying various proportion of the G8969>A mutation. (**A**) Percentages of the G8969>A mutation in cybrid cell lines. (**B**) Mitochondrial ultrastructure (magnification ×50,000). In C-98, arrows point to aberrant, torus and horseshoe-shape, mitochondria. (**C**) Cellular respiration. The OCR (oxygen consumption rates) were evaluated in cybrids with different levels of G8969>A mutation. OSR (oligomycin-sensitive respiration), BR (basal respiration) and MR (maximal respiration). *Stands for comparison to WT, # is comparison to C-19. (**D**) Levels of reactive oxygen species (ROS). The levels of ROS were assessed using 2′,7′-dichlorodihydrofluorescein diacetate (DCFH-DA)^47^. Immunofluorescence was visualized by confocal microscopy (left panel) and quantified by flow cytometry and expressed as decimal logarithm versus cell number (right panel). WT, control cells; C-19, C-67, and C-98 are cybrid cell lines containing 19%, 67% and 98% of the G8969>A mutation, respectively. ρ^0^, cells lacking mtDNA.

To further define the pathogenesis of the G8969>A mutation, cybrid cell lines containing various proportions of this mutation (19%, C-19; 67%, C-67; and 98%, C-98) were constructed (Fig. 3A). This is a common strategy used to ascertain that a disease is linked to mitochondrial DNA^10^. Increasing the G8969>A mutation load was paralleled by changes in the ultrastructure of mitochondria, with the appearance of torus-like and horseshoe-shape mitochondria with only a few or a single cristae present (Fig. 3B). Oxygen consumption was decreased in the mutated cybrid cells lines and the residual respiration was poorly inhibited by oligomycin, a specific inhibitor of ATP synthase (Fig. 3C). Respiration was also decreased in the mutant cybrid cells compared to the control in the presence of the proton ionophore carbonylcyanide p-trifluoromethoxyphenylhydrazone (FCCP), indicating a reduced content of some of the enzymes involved in electron transfer to oxygen. Finally, increasing the mutation load was paralleled by higher levels of reactive oxygen species (ROS) (Fig. 3D). All these defects are typically observed in cells with a defective ATP synthase^11^,^12^,^13^,^14^,^15^,^16^.

### Consequences of the G8969>A mutation in yeast

**Figure 4:**
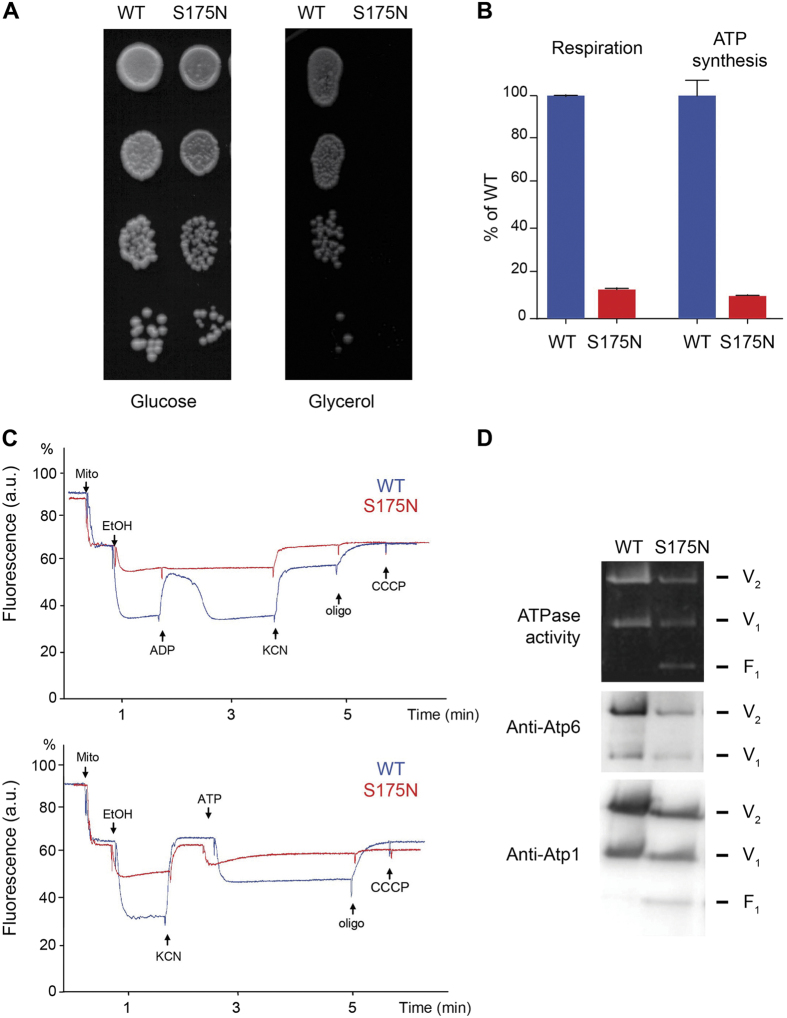
Consequences of an equivalent of the G8969>A mutation (atp6-S175N) in yeast. (**A**) *WT* and *atp6*-S175N mutant yeast cells grown in glucose were serially diluted and spotted onto plates containing glucose or glycerol; photographs were taken after 4 days of incubation. (**B**) Oxygen consumption (state 3) and ATP synthesis rates measured with NADH as an electron donor, expressed in % of the WT (see Supplementary Table S4 for details). (**C**) Variations in mitochondrial membrane potential (ΔΨ). These experiments were performed using Rhodamine 123, a fluorescent cationic dye. Increasing ΔΨ is followed by the uptake of the dye inside the matrix space and concomitant fluorescence quenching. The tracings in the upper panel reveal the capacity of externally added ADP to induce ΔΨ consumption after energization of the mitochondrial inner membrane by the respiratory chain (WT in blue, the mutant in red). The tracings in the bottom directly reflect the proton-pumping activity of ATP synthase using externally added ATP, after inhibition of the respiratory chain EtOH, ethanol; Oligo, oligomycin; Mito, mitochondria. (**D**) BN-PAGE analysis of mitochondrial protein extracts solubilized with 2% digitonin. ATP synthase complexes (V_2_, dimeric; V_1_, monomeric) and free F_1_ particles were visualized in-gel by their ATPase activity and by western blotting after transfer onto nitrocellulose using antibodies against subunits Atp1p (α-F_1_) and Atp6p (subunit-*6*).

To understand how the G8969>A mutation molecularly impacts ATP synthase, a yeast model of this mutation was created. The S148 residue of the human *a*-subunit corresponds to S175 of the yeast protein (referred to as subunit-*6*). As yeast is unable to stably maintain heteroplasmy^17^, it was possible to generate homoplasmic strains in which all the mtDNA molecules contain the *atp6*-S175N mutation, which enabled a detailed investigation of the functional consequences of this mutation. We found that the *atp6*-S175N mutation virtually abolished the growth of yeast on non-fermentable carbon sources like glycerol (Fig. 4A), conditions under which the presence of a functional ATP synthase is absolutely essential. To determine the impact of the *atp6*-S175N mutation on oxidative phosphorylation, mitochondria were isolated from the cells grown in rich galactose medium and analyzed as described hereafter.

#### Respiration

We first measured mitochondrial oxygen consumption using NADH as an electron donor, alone (basal, state 4 respiration), after further addition of ADP (state 3, phosphorylating conditions) or in the presence of the membrane proton ionophore CCCP (carbonyl cyanide *m*-chlorophenylhydrazone) (uncoupled respiration) (Fig. 4B, Supplementary Table S4). In all these conditions, respiration was strongly decreased in the *atp6*-S175N mutant, by about 90% with respect to the WT. These findings were an indication that the mutation severely compromised ATP synthase. Indeed yeast mutants with a defective ATP synthase often respire poorly^18^,^19^,^20^,^21^,^22^.

#### ATP synthesis/hydrolysis

We measured the rate of mitochondrial ATP synthesis using NADH as a respiratory substrate and in the presence of a large excess of external ADP. In these conditions, ATP is synthesized only by ATP synthase using the proton-motive force generated by complexes III and IV (there is no complex I in *S. cerevisiae*). The rate of ATP synthesis was reduced in the *atp6*-S175N mutant by 90% compared with the *WT* (Fig. 3B, Supplementary Table S4). Since state 3 respiration and ATP synthesis rates were decreased in similar proportions in the mutant, it can be inferred that the observed oxidative phosphorylation deficit mostly resulted from a slower ATP synthesis rate rather than a less efficient coupling of the mitochondrial energy transducing system.

Though the ATP synthesis activity of ATP synthase was considerably affected by the atp6-S75N mutation, its ATP hydrolytic activity was only partially compromised, as evidenced by direct measurements on total mitochondrial samples (Supplementary Table S4) and in BN-gels loaded with mitochondrial extracts solubilized with digitonin (Fig. 4D, see below). However, while this activity was largely inhibited by oligomycin in the *WT*, it was mostly insensitive to this drug in the mutant, which is frequently observed in yeast F_O_-deficient mutants^14^,^18^,^19^,^23^,^24^.

#### Mitochondrial membrane potential

The consequences of the *atp6*-S175N mutation on oxidative phosphorylation in yeast cells were analysed further using Rhodamine 123. This is a fluorescent cationic dye that can be used to monitor changes in the inner membrane potential (ΔΨ) on intact mitochondria^25^. Increasing ΔΨ is followed by the uptake of the dye inside the matrix space and concomitant fluorescence quenching. In a first set of experiments (Fig. 4C, upper panel), we tested the capacity of externally added ADP to induce ΔΨ consumption. To this end, the mitochondria were energized first by the respiratory chain, with electrons from ethanol. Due to their reduced capacity to respire (Fig. 4B, Supplementary Table S4), *atp6*-S175N mitochondria were poorly energized in comparison to the *W*T. Normally, further addition of small amounts of ADP induces a transient fluorescence increase due to ΔΨ consumption by the ATP synthase during phosphorylation of the added ADP. This was indeed observed in the *WT*, whereas *atp6*-S175N mitochondria were virtually insensitive to ADP. KCN was then added to inhibit complex IV, which, in *WT* mitochondria, resulted in a partial ΔΨ collapse. The remaining potential is due to the pumping of protons by the F_0_ component of ATP synthase coupled to the hydrolysis by its F_1_ sector of the ATP that accumulated in the mitochondrial matrix during phosphorylation of the added ADP. Indeed, this potential was lost upon further addition of oligomycin. No oligomycin-sensitive ΔΨ was observed in the mutant mitochondria, which further reflected their strongly reduced capacity to produce ATP.

In another set of experiments, we directly tested the proton-pumping activity of ATP synthase using externally added ATP, independently of the respiratory chain (Fig. 4C, bottom panel). The mitochondria were first energized with ethanol to remove the natural inhibitory peptide (IF1) of the F_1_-ATPase. The mitochondrial membrane potential was then collapsed with KCN, and less than one minute later, thus well before IF1 rebinding^26^, ATP was added. Normally, the external ATP is counter-exchanged against ADP present in the matrix by the ADP/ATP translocase, which does not require any ΔΨ, and the ATP can then be hydrolyzed by F_1_ coupled to F_O_-mediated proton transport out of the matrix. The ATP addition promoted in *WT* mitochondria a large and stable fluorescence quenching of the dye that was reversed upon F_0_ inhibition with oligomycin, whereas *atp6*-S175N mitochondria were mostly insensitive to ATP, indicating that the mutation inactivates the proton channel of ATP synthase.

#### Assembly/stability of ATP synthase

Blue-Native-Polacrylamide-Gel-Electrophoresis (BN-PAGE) analyses of mitochondrial proteins extracted with digitonin revealed that full ATP synthase complexes accumulated in *atp6*-S175N mitochondria as monomeric and dimeric units, although in lesser amounts than in the *WT* (Fig. 4D). The drop in fully assembled ATP synthase (about 50% *vs* the WT) was less important than the drop (90%) in ATP synthesis activity (see above). It can be inferred that the remaining assembled F_1_F_0_ complexes are mostly unable to produce ATP, indicating that the *atp6*-S175N mutation blocks the ATP synthase proton channel. Free F_1_ particles, almost absent in the *WT*, were detected in the mutant, which indicates that the *atp6*-S175N mutation also partially compromised the assembly/stability of the F_0_ component of ATP synthase.

### Topological location of the S148 residue of human a-subunit in the inner mitochondrial membrane

**Figure 5:**
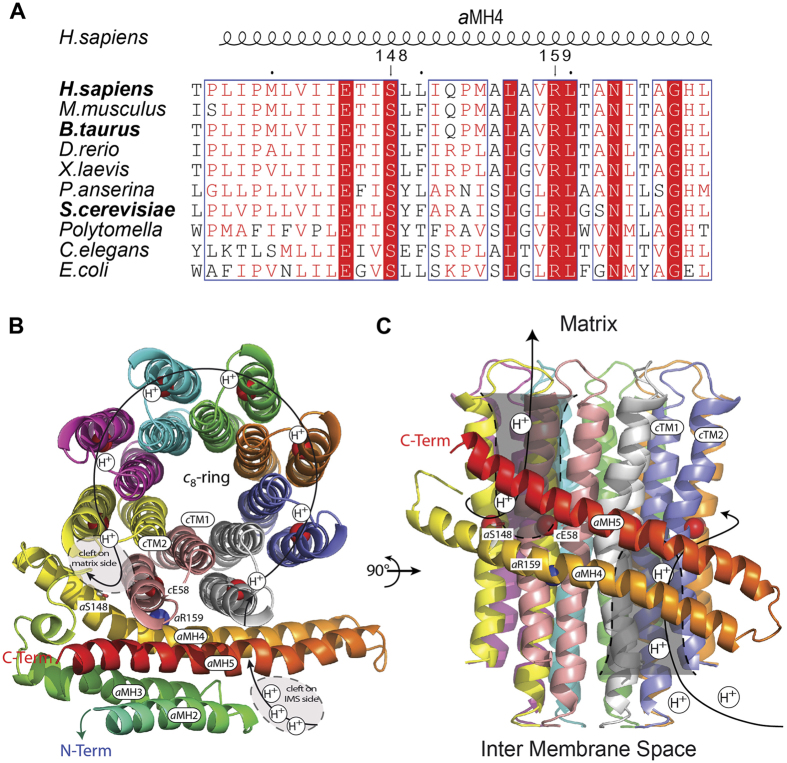
Topological location of the aS148 residue of a-subunit of human ATP synthase in the inner mitochondrial membrane. (**A**) Alignment of *a*-subunits from mitochondrial and bacterial origins. The G8969>A mutation leads to the replacement with asparagine of the evolutionary conserved serine at position 148 of the human *a*-subunit. (**B,C**) Structural homology model of human *c*_8_-ring (PDB # 2XND) in complex with four helical segments of the *a*-subunit (*a*MH2-5), coloured from blue (N-Term) to red (C-Term) viewed from the matrix (**B**) and from the membrane plane (**C**). The side chain of *a*S148 residue is shown as sticks, the *c*E58 and *a*R159 are as spheres. The arrowed line indicates the pathway for protons during ATP synthesis. Protons enter and exit the *c*-ring/*a*-subunit through hydrophilic clefts in the membrane represented by the grey areas surrounded by dashed lines^31^.

The *a*-subunit and a ring of *c*-subunits are responsible for the transport of protons across the membrane domain (F_0_) of ATP synthase^27^,^28^. This transport drives the rotation of the c-ring, which results in conformational changes in the catalytic domain (F_1_) of the enzyme that ultimately promote ATP synthesis. Recent cryo-electron microscopy analyses of ATP synthase from mammals, fungi and bacteria have revealed four horizontal membrane-intrinsic-helices (MH) in the *a*-subunit that run along the *c*-ring^29^,^30^,^31^. Based on these highly conserved structures, we construct a structural homology model of this 4-helix bundle for the human a-subunit (referred to as aMH2–5) in interaction with a c8-ring (Fig. 5B,C)^32^. At the interface between *a*-subunit and the *c*-ring, near the middle of the membrane, are two electrically charged residues (aR159 and cE58) that are well known to be involved in the transfer of protons mediated by ATP synthase^27^,^28^. A hydrophilic cleft on the external side of the inner membrane is presumed to enable protons to reach cE58 from the inter membrane space and, after a c-ring rotation, the protons are supposed to be released into a second hydrophilic cleft on the matrix side of the membrane (Fig. 5B,C)^31^. The *a*S148 residue mutated in our patient is in the matrix-localized cleft, near the catalytic *c*E58 residue. Below we discuss the possibility that the *a*S148 residue helps, through its hydroxyl group, the exit of protons from the c-ring int the mitochondrial matrix.

## Discussion

Our study reports the case of a patient initially diagnosed with isolated common IgA nephropathy followed by brain and muscle problems, a clinical presentation typically observed in mitochondrial cytopathies^2^,^3^,^33^,^34^. We thus came to suspect a possible involvement of mitochondria in the disease process, which was supported by renal tissues analyses showing an enhanced fragmentation of the mitochondrial network (Fig. 1D) and diminished NADH oxidation and cytochrome *c* oxidase activities (Fig. 1E), and there was a strong accumulation of lactate in cerebrospinal fluid from the patient (Supplementary Fig. S2).

These findings prompt us to sequence the mtDNA of the patient, which revealed a point mutation (G8969>A) in the *ATP6* gene (Fig. 2). As a result, a serine residue in the *a*-subunit of ATP synthase, strictly conserved in mitochondria and bacteria (Fig. 5A), is replaced by asparagine. The use of cybrid cell lines with a controlled nuclear background enabling a proper expression of mitochondrial function provided strong evidence that the G8969>A mutation is very detrimental to ATP synthase. Indeed, increasing the mutation load in these cells was paralleled by the appearance of abnormal mitochondrial morphologies (Fig. 3B), a reduced rate of oxygen consumption (Fig. 3C) and increased production of ROS (Fig. 3D), which are all typical consequences of defects in the ATP synthase^11^,^13^. Furthermore, we found that in a homoplasmic yeast model of the G8969>A mutation (*atp6*-S175N) ATP synthase proton translocation was very severely compromised. In our structural homology model of the human *a*-subunit/c-ring complex (Fig. 5B), the mutated serine residue in a hydrophilic pocket on the matrix side of the membrane that has been proposed to provide a pathway for protons through the F_0_^31^. This suggests that the mutated serine has possibly a role in F_0_-mediated proton translocation, owing to the presence on its lateral side chain of a hydroxyl group that has the potential to exchange protons. However, mutating this residue in *E. coli* (*a*S199) into alanine, an aliphatic amino acid residue unable to conduct protons, had no apparent effects on ATP synthase function^35^. Thus, *a*S148 of human *a*-subunit is likely not an obligate component of the ATP synthase proton channel, but affects proton translocation indirectly.

The strong abundance (89%) of the G8969>A mutation in the kidney compared to other tissues from the patient (Fig. 3D) is likely the reason why this organ was primarily affected. Indeed, with such a deleterious mutation and only 10% of wild type mtDNA, there is no doubt that the kidney, an organ with high energy demands^36^, was in the patient deprived of enough ATP to function normally. The presence of the same mutation in the mtDNA from the mother suggests a maternal inheritance. The lack of symptoms in the mother can be explained by a lower content of the mutation, about 60%, which is typically below the threshold at which mtDNA mutations become detrimental for human health^8^. The enhanced production of ROS induced by the G8969>A may also contribute to the disease process by damaging cellular constituents and depolarizing the inner mitochondrial membrane, which can ultimately lead to apoptosis^37^,^38^.

The *ATP6* gene has already been implicated in several human disorders, such as the Leigh Syndrome (LS), bilateral striatal lesions of childhood (BSLC), neuropathy ataxia, and retinitis pigmentosa (NARP)^11^,^39^,^40^. The G8969>A mutation was recently found in a patient presenting with MLASA, which is a rare mitochondrial disorder characterized by developmental delay, sensorineural hearing loss, epilepsy, agenesis of the corpus callosum, failure to thrive and stroke-like episodes^41^. The present study extends the spectrum of diseases related to the mitochondrial *ATP6* gene, and provides the first successful discovery of a defined genetic locus contributing to a disease that was primarily diagnosed as IgA nephropathy. There is no known case of a mitochondrial cytopathy that manifested first as an isolated kidney disorder^6^. The differences in clinical presentation between the patient displaying MLASA and the case reported in our study are most likely due to distinct distributions of the G8969>A mutation within cells and tissues.

Immunosuppressive treatment is commonly used in renal diseases, and corticosteroids have been used for over 20 years for treating IgA nephropathy. Our patient was therefore treated with steroids while her mitochondrial dysfunction was still unknown, and she showed a complete remission. Finsterer J *et al*.^42^ did report a mitochondriopathy case without kidney impairment, in which high doses of corticosteroids were proven to be beneficial by an as-yet-unknown mechanism. Our work further supports that the use of corticosteroids might be beneficial against mitochondriopathies, which is an interesting observation.

IgAN is one the most common renal diseases, which is a pattern of glomerular injury defined by the presence of glomerular IgA deposits in immunofluorescence microscopy with various non-specific histopathological lesions^43^. As such, IgAN is not a disease diagnosis; rather, it is a descriptive term for a certain type of glomerular damage. In the absence of family history and whole exome or genome sequencing a direct causation in kidney disease cannot be firmly ascribed to the G8969>A mutation. Even though we cannot preclude that the IgAN presentation of our patient possibly involves or is caused by another yet unknown pathogenic factor of nuclear DNA origin, we believe that our study is interesting to the field of kidney diseases. Indeed the strong abundance in the kidney of the patient of a mtDNA mutation that severely compromises mitochondrial function possibly contributed or aggravated the renal failure. Such a case has not been described before. Based on the findings reported in this study, and results obtained with other patients in which a relatively high prevalence of mtDNA mutations and mitochondrial morphology defects were observed (manuscript in preparation), we suggest that nephrologists should pay more attention on the possible involvement of a mitochondrial dysfunction when evaluating patients suffering from kidney problems and report more systematically for the presence/absence of abnormal mitochondrial structures when electron microscopy in renal biopsy samples is performed.

## Methods

### Subjects

We ascertained a female Chinese patient with recurrent kidney disease and multiple systemic dysfunctions through National Clinical Research Center of Kidney Diseases, Jinling Hospital, Nanjing University School of Medicine, Nanjing, China. One hundred healthy control adults were recruited from a panel of unaffected, genetically unrelated Han Chinese individuals from the same geographic region. Written informed consent was obtained from the patient and her mother as well as the 100 healthy controls. All experimental protocols were approved by the Ethics Committee of the Jinling Hospital (Nanjing, China) in accordance with the Declaration of Helsinki. All methods and procedures were conducted according to the manufacturers’ instructions or in strict accordance with the recommendations in the guidelines set forth by the Ethics Committee of the Jinling Hospital (Nanjing, China).

### Clinical and histological evaluation

Evaluations of the patient were taken using different methods of assessment, including medical history, physical examination and laboratory tests (routine urine and blood test) every time she visited the clinic. Renal biopsy was performed under ultrasound guidance by an experienced investigator, and tissues were processed for light, electron, and immunofluorescence microscopy as described^44^. Electron microscopy examination was conducted with 60–80 nm ultrathin tissue sections fixed in 2.5% glutaraldehyde and embedded in epon. Mitochondrial enzyme activity analysis was carried out on frozen renal tissue by histochemistry staining for cytochrome c oxidase (COX) and nicotinamide adenine dinucleotide (NADH) dehydrogenase as described^45^.

### Mitochondrial genome analyses

Total genomic DNA was isolated from blood, urine sediments and renal samples using the QIAamp DNA extraction kit (QIAGEN). The 16.5 kb mtDNA was amplified using 15 polymerase chain reaction (PCR) primer pairs and then sequenced. The sequencing data were compared against the revised Cambridge Reference Sequence (rCRS) of human mtDNA (GenBank NC_012920.1). For detection of mtDNA deletion, long range PCR was amplified using two primer pairs and the amplified DNA fragments were separated by electrophoresis on a 0.8% agarose gel. Real-time quantitative PCR was used to determine mtDNA copy number, relative to the nuclear 18S rRNA gene, using two primers specific of the mitochondrial COX1 locus and two primers specific of the nuclear 18SrRNA locus. The Pyromark Q24 platform (Qiagen) was used to assess the levels of heteroplasmy of the G8969>A mutation. For this, a fragment around position 8969 was PCR amplified with two primers firstly; then pyrosequencing of the amplificate was performed using the sequencing primer. The presence of the G8969>A mutation was also analyzed in 100 controls. All the primers used in this study were listed in Supplementary Table S1.

### Transmitochondrial cybrid construction and analyses

Transmitochondrial cybrids were generated on a background of HeLa cell line devoid of their own mtDNA (ρ^0^ cells, a gift of Professor Bin Lu). Platelets of the patient were extracted from 8~10 ml peripheral blood and subsequently fused with ρ^0^ cells using 50% (w/v) polyethylene glycol 1500 solution (Fluka) essentially as described^46^. A series of remarkably stable clones harboring 19%, 67% and 98% of the G8969A mutant mtDNA were obtained. HeLa cells and cybrids were cultured in Dulbecco’s modified Eagle’s medium containing 10% fetal bovine serum and 100 units/ml of penicillin streptomycin. For growing ρ^0^ cells, the medium was supplemented with sodium pyruvate (110 mg/ml) and uridine (50 mg/ml). Electron microscopy analyses were performed using cells fixed with 2.5% (v/v) glutaraldehyde in epon.

Oxygen consumption rates (OCR) were measured using the Seahorse XF96 Extracellular Flux Analyser (Seahorse Bioscience, North Billerica, MA, USA). 8.0 × 103 of cybrid cells, HeLa and ρ^0^ cells were seeded into 96 well Seahorse microplates in 80 μl of growth medium and incubated at 37 °C in 5% CO2 for 24 h and the calibrator plate was equilibrated in a non-CO2 incubator overnight. Basal respiration (BR) corresponds to the drop in OCR induced by 1 μM antimycin. The part of BR insensitive to 1 mM oligomycin corresponds to OSR. Maximal respiratory (MR) was induced by uncoupling the mitochondrial membrane 1 μM proton ionophore carbonylcyanide p-trifluoromethoxyphenylhydrazone (FCCP). The levels of ROS production was assessed by staining with 2′,7′-dichlorodihydrofluorescein diacetate (DCFH-DA) as described^47^. Immunofluorescence green staining was observed under confocal microscopy and fluorescence intensity was measured by flow cytometry (excitation at 488 nm and emission at 529 nm) and is expressed as decimal logarithm versus cell number.

### Construction of the yeast atp6-S175N mutant

The QuikChange XL Site-directed Mutagenesis Kit of Stratagene was used to introduce a G8969>A mutation equivalent to the yeast *ATP6* gene cloned in pUC19^18^, using the primer 5′-CCTTTATTAGTTATTATTGAAACTTTAAATTATTTCGCTAGAGCTATTTCATTAGG. The mutated (atp6-S175N) gene was cloned into plasmid pJM2 containing the yeast mitochondrial COX2 gene as a mitochondrial transformation marker^48^. The resulting plasmid (pRK66) was introduced by co-transformation with the nuclear selectable LEU2 plasmid Yep351 into the ρ^0^ strain DFS160 (see Supplementary Table S2 for complete genotype) using the biolistic PDS-1000/He particle delivery system (Bio-Rad). Mitochondrial transformants (RKY104) were identified among the Leu+ clones by their ability to restore respiration when mated to NB40-3C, a strain that bears a deletion in the mitochondrial COX2 gene^48^. They were then crossed to the atp6::ARG8m deletion strain MR10^18^, which produced clones (RKY105) harboring the MR10 nucleus and where the ARG8m open reading frame had been replaced with the mutated atp6-S175N gene; these clones were identified as arginine prototrophs and their inability to grow on non-fermentable carbon source. DNA sequencing of RKY105 revealed no other change in the *ATP6* gene than the atp6-S175N mutation.

### Biochemical analyses of yeast mitochondria

Mitochondrial enzyme assays and membrane potential analyses were performed on mitochondria isolated from yeast cells grown in rich galactose (YPGALA). The mitochondria were prepared as described^49^. Oxygen consumption rates were measured with a Clark electrode in 0.65 M mannitol, 0.36 mM EGTA, 5 mM Tris/phosphate, 10 mM Tris/maleate pH 6.8 (respiration buffer), as described previously^50^.

Variations in transmembrane potential (ΔΨ) were evaluated in the respiration buffer by measurement of rhodamine 123 fluorescence quenching with a Cary Eclipse Fluorescence Spectrophotometer (Agilent Technologies, Santa Clara, CA, USA)^25^,^51^. For ATP synthesis rate measurements, the mitochondria (0.15 mg/ml) were placed in a 1 ml thermostatically controlled chamber at 28 °C in respiration buffer. The reaction was started by the addition of 4 mM NADH and 1 mM ADP and stopped by 3.5% perchloric acid, 12.5 mM EDTA. Samples were neutralized to pH 6.5 by addition of KOH, 0.3 M MOPS and ATP was quantified by Kinase-Glo Max Luminescence Kinase Assays (Promega) on a Beckman Coulter's Paradigm Plate Reader. Part of the ATP produced by the F_1_F_2_-ATP synthase was assessed using oligomycin (20 μg/mg of proteins).

BN–PAGE analysis was performed as described^52^. Polyclonal antibodies raised against yeast ATP synthase were used after 1:10000 dilution. Nitrocellulose membranes were incubated with peroxidase-labeled antibodies at a 1:10000 dilution and revealed with the ECL reagent of Millipore.

### Multiple sequence alignment and homology modelling of human ac_8_ complex

Multiple sequence alignment of ATP synthase a-subunit was performed using Clustalw. TMHMM 2.0 and PSIPRED 3.3 servers were used to predict transmembrane segments and secondary structures, respectively. Figure 5A was drawn using the ESPRIPT program. The homology model of human ac8 complex is based on the atomic model build in the cryo-electron microscopy density map of the bovine ATP synthase (pdb:5ARH; emdb:3166)^29^ and the bovine c8-ring crystal structure (pdb:2xnd)^32^. It was built using Phire2, energy minimized using Phenix, Fig. 5B,C was drawn using Pymol.

### Statistics

Data are expressed as means ± S.D. or S.E.M. SPSS software (16.0), unpaired t test and analysis of variance (ANOVA) were used for comparison between groups of data. A p value < 0.05 was considered as statistically significant.

## Additional Information

**How to cite this article**: Wen, S. *et al*. Identification of G8969>A in mitochondrial *ATP6* gene that severely compromises ATP synthase function in a patient with IgA nephropathy. *Sci. Rep*. **6**, 36313; doi: 10.1038/srep36313 (2016).

**Publisher’s note:** Springer Nature remains neutral with regard to jurisdictional claims in published maps and institutional affiliations.

## Supplementary Material

Supplementary Information
