# Supplementary material for: Identification of G8969>A in mitochondrial ATP6 gene that severely compromises ATP synthase function in a patient with IgA nephropathy
Source: Sci Rep. 2016 Nov 4;6:36313. doi: 10.1038/srep36313 (PMC5095641; doi:10.1038/srep36313)
Supplement: Supplementary Information [file srep36313-s1.pdf]

Supplementary information

Identification of G8969>A in mitochondrial *ATP6* gene that severely compromises ATP synthase function in a patient with IgA nephropathy

Shuzhen Wen[1#], Katarzyna Niedzwiecka[2#], Weiwei Zhao[1], Shutian Xu[1], Shaoshan Liang[1], Xiaodong Zhu[1], Honglang Xie[1], Déborah Tribouillard-Tanvier[3,4,5], Marie-France Giraud[3,4], Caihong Zeng[1], Alain Dautant[3,4], Róża Kucharczyk[2], Zhihong Liu[1*], Jean-Paul di Rago[3,4*], and Huimei Chen[1*]

- **Supplementary Table S1** Primers used in this study
- **Supplementary Table S2** Genotypes and sources of yeast strains
- **Supplementary Table S3** List of the mtDNA nucleotide changes in the patient relative to the reference sequence of the human mitochondrial genome
- **Supplementary Table S4** Influence of the *atp6*-S175N mutation on yeast mitochondrial respiration, ATP synthesis and hydrolysis in yeast mitochondria.

- **Supplementary Figure S1** Quantification of COX and nicotinamide adenine dinucleotide (NADH) dehydrogenase activities in renal biopsy samples.
- **Supplementary Figure S2**. Lactate content is increased in cerebrospinal fluid from the patient.

Supplementary Table S1 Primers used in this study

A) For detection of mtDNA point mutations

N°	Forward	Reverse	PCR product size (bp)
1	CCCACAGTTTATGTAGCTTACC	GTACTATATCTATTGCGCCAG	1215
2	ACTACCAGACAACCTTAGCC	AACATCGAGGTCGTAAACCC	1293
3	CTTCACCAGTCAAAGCGAAC	AGAAGTAGGGTCTTGGTGAC	1242
4	CGAACTAGTCTCAGGCTTCAAC	TCGTGGTGCTGGAGTTTAAG	1228
5	ACGTAAGCCTTCTCCTCACT	TCGTTACCTAGAAGGTTGCC	1138
6	CCGACCGTTGACTATTCTCT	GATGGCAAATACAGCTCCTA	1160
7	GCAAACTCATCACTAGACATCG	AGCTTTACAGTGGGCTCTAG	1329
8	ACCACAGTTTCATGCCCATC	TGGCCTTGGTATGTGCTTTC	1223
9	CACTTCCACTCCATAACGCT	GTTGAGGGTTATGAGAGTAGC	1308
10	TACCAAATGCCCCTCATTTA	GTAATGAGGATGTAAGCCCG	1272
11	TTCAATCAGCCACATAGCCC	GATGAAACCGATATCGCCGA	1260
12	GAGGGCGTAGGAATTATATCC	GTCAGGTTAGGTCTAGGAGG	1240
13	CATACTCGGATTCTACCCTAG	TGTAATTACTGTGGCCCCT	1280
14	TCGGCATTATCCTCCTGCTT	GTGCTATGTACGGTAAATGGC	1250
15	TGACTCACCCATCAACAACC	ATAGAAAGGCTAGGACCAAACC	1179

B) For detection of mtDNA deletion

N°	Forward	Reverse	PCR product size (bp)
1	GCACCCTATGTCGCAGTATCTGTCTTTG	GGACGAGAAGGGATTTGACTGTAATGTGC	16255
2	CACTTCCACTCCATAACGCTCCTCATACT	GGGCTATTGGTTGAATGAGTAGGCTGATG	16250

C) For detection of mtDNA copy number

COX1	TTCGCCGACCGTTGACTATTCTCT	AAGATTATTACAAATGCATGGGC	197
18S	GTCTGTGATGCCCTTAGATG	AGCTTATGACCCGCACTTAC	177

D) For pyrosequencing of G8969>A mutation

amplification primers	TCTTACCACAAGGCACACC	AAGGCGACAGCGATTTCTA	241
sequencing primer	ATTGGTTGAATGAGTA		

Supplementary Table S2 Genotypes and sources of yeast strains

**Strain**	**Nuclear genotype**	**mtDNA**	**Source**
DFS160	*MAT**α** leu2Δ ura3-52 ade2-101 arg8::URA3 kar1-1*	ρ^0	[1]
NB40-3C	*MAT**a** lys2 leu2-3,112 ura3-52 his3ΔHinDIII arg8::hisG*	ρ^+ *cox2-62*	[1]
MR6	*MAT**a** ade2-1 his3-11,15 trp1-1 leu2-3,112 ura3-1 arg8::HIS3*	ρ^+	[2]
MR10	*MAT**a** ade2-1 his3-11,15 trp1-1 leu2-3,112 ura3-1 arg8::HIS3*	ρ^+ *atp6::ARG8*^{m}	[2]
RKY104	*MAT**α** leu2Δ ura3-52 ade2-101 arg8::URA3 kar1-1*	ρ^- *atp6-S175N*	This study
RKY105	*MAT**a** ade2-1 his3-11,15 trp1-1 leu2-3,112 ura3-1 arg8::HIS3*	ρ^+*atp6-S175N*	This study

Supplementary Table S3 List of the mtDNA nucleotide changes in the patient relative to the reference sequence of the human mitochondrial genome (http://www.mtdb.igp.uu.se/).

Locus	Nucleotide position	Nucleotide and a.a. changes [a]	Conservation [b]	Frequency [c]	Disease
D-Loop	73	A to G		1555/1865	
	152	T to C		396/1865	
	214	A to G		12/1865	
	249*	-A		103/1865	
	263	A to G		1861/1865	
	315	+C			
	489*	T to C		777/2144	
	16185*	C to T		29/1867	
	16223	C to T		875/1867	
	16260*	C to T		37/1867	
	16298*	T to C		169/1867	
12S rRNA	709	G to A	G/A/A/A	444/2704	
	750	A to G	A/G/A/A	2682/2704	
	1438	A to G	A/T/T/G	2620/2704	Diabetes [3]
16S rRNA	2706	A to G	A/A/A/G	2178/2704	
	3221	A to G	A/C/A/T	7/2704	
ND2	4715*	A to G		84/2704	
	4769	A to G		2674/2704	
	4853	G to A		2/2704	
	5147	G to A		135/2704	
	5424	C to T (H>T)	H/L/K/S		
CO1	6752*	A to G		44/2704	
	7028	C to T		2199/2704	
ATP6	7196*	C to A		84/2704	
	8584*	G to A (A>T)	A/V/V/I	129/2704	
	8701	A to G (T>A)	T/S/L/Q	933/2704	Lung cancer [4]
	8860	A to G (T>A)	T/A/A/T	2698/2704	
	**8969**	**G to A (S>N)**	**S/S/S/S**	**0/2704**	**MLASA [5]**
	9090*	T to C		31/2704	
CO3	9540	T to C		944/2704	
ND3	10208	T to C		2/2704	
	10398	A to G (T >A)	T/T/T/A	1242/2704	PD [6]
	10400*	C to T		724/2704	
ND4	10873	T to C		943/2704	
	11719	G to A		2100/2704	
ND5	12705	C to T		1223/2704	
CYB	14766	C to T (T>I)	T/T/S/S	610/2704	
	14783*	T to C		720/2704	
	15043*	G to A		777/2704	
	15301	G to A		867/2704	
	15326	A to G (T>A)	T/I/M/I	2687/2704	
	15487*	A to T		83/2704	
	15784*	T to C		85/2704	

[a]In brackets are the corresponding amino acid changes; [b]Conservation of amino acid in polypeptides and nucleotides in rRNAs in human (H), bovine (B), mouse (M), and Xenopus laevis (X); [c]Frequency refers to the occurrence of the detected nucleotide changes in 2704 normal individuals. *Stands for haplogroup Z3 nucleotide changes. Nucleotide changes that have been associated to disease (last column) are indicated.

Supplementary Table S4. Influence of the *atp6*-S175N mutation on respiration, ATP synthesis and hydrolysis in yeast mitochondria.

		**28°C**		**36°C**	
		MR6 (WT)	RKY105 (S175N)	MR6 (WT)	RKY105 (S175N)
Respiration rates (nmol O.min^{-1}.mg^{-1})	NADH	543 ± 1	60 ± 5	241 ± 9	37 ± 7
	NADH+ADP	1051 ± 59	60 ± 4	638 ± 48	40 ± 3
	NADH+CCCP	1665 ± 87	86 ± 9	1065 ± 18	55 ± 8
	Asc/TMPD+ CCCP	2772 ± 337	1064 ± 250	1609 ± 30	651 ± 97
ATP synthesis rate (nmol Pi.min^{-1}.mg^{-1})	- oligo	1620 ± 78	164 ± 9	1271 ± 79	144 ± 10
	+ oligo	223 ± 53	60 ± 10	194 ± 10	95 ± 9
$\rho^{0/-}$(%)		<5	<5	37 ±3	55 ± 9
ATPase activity (µmol Pi.min^{-1}.mg^{-1})	- oligo	3.5 ± 0.6	1.75 ± 0.61	2.00 ± 0.32	1.02 ± 0.25
	+oligo	0.39 ± 0.03	1.39 ± 0.39	0.6 ± 0.05	0.96 ± 0.20

Note: Mitochondria were isolated from cells strains grown for 5-6 generations in YPGALA medium (rich galactose) at 28°C or 36°C. Reaction mixes contained 0.15 mg/ml protein, 4 mM NADH, 150 (for respiration assays) or 750 (for ATP synthesis) µM ADP, 12.5 mM ascorbate (Asc), 1.4 mM N,N,N,N,-tetramethyl-p-phenylenediamine (*TMPD*), 4 µM CCCP, 3 µg/ml oligomycin (oligo). The values reported are averages of triplicate assays ± standard deviation. Respiratory and ATP synthesis activities were measured using freshly isolated, osmotically protected mitochondria buffered at pH 6.8. For ATPase assays, mitochondria kept at –80°C were thawed and the reaction performed in absence of osmotic protection and at pH 8.4.

Supplementary Figure S1. Quantification of COX and nicotinamide adenine dinucleotide (NADH) dehydrogenase activities in renal biopsy samples.

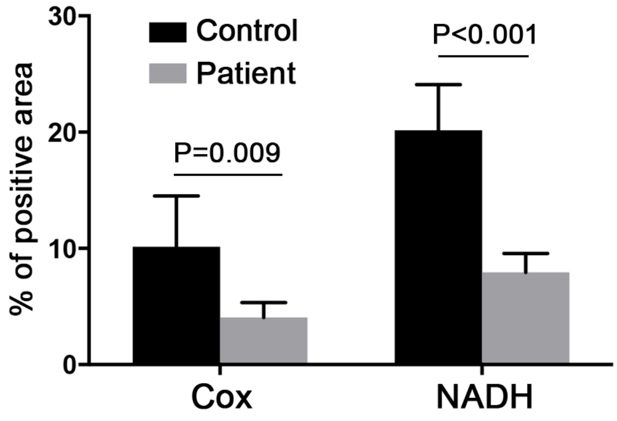

Supplementary Figure S2. Lactate content in cerebrospinal fluid from the patient. 1H magnetic resonance spectroscopy (MRS) spectrum obtained (MRS) showing singlet resonance from Nacetylaspartate (NAA, 2.0 ppm), and the lactate doublet (lac, 1.3 ppm).

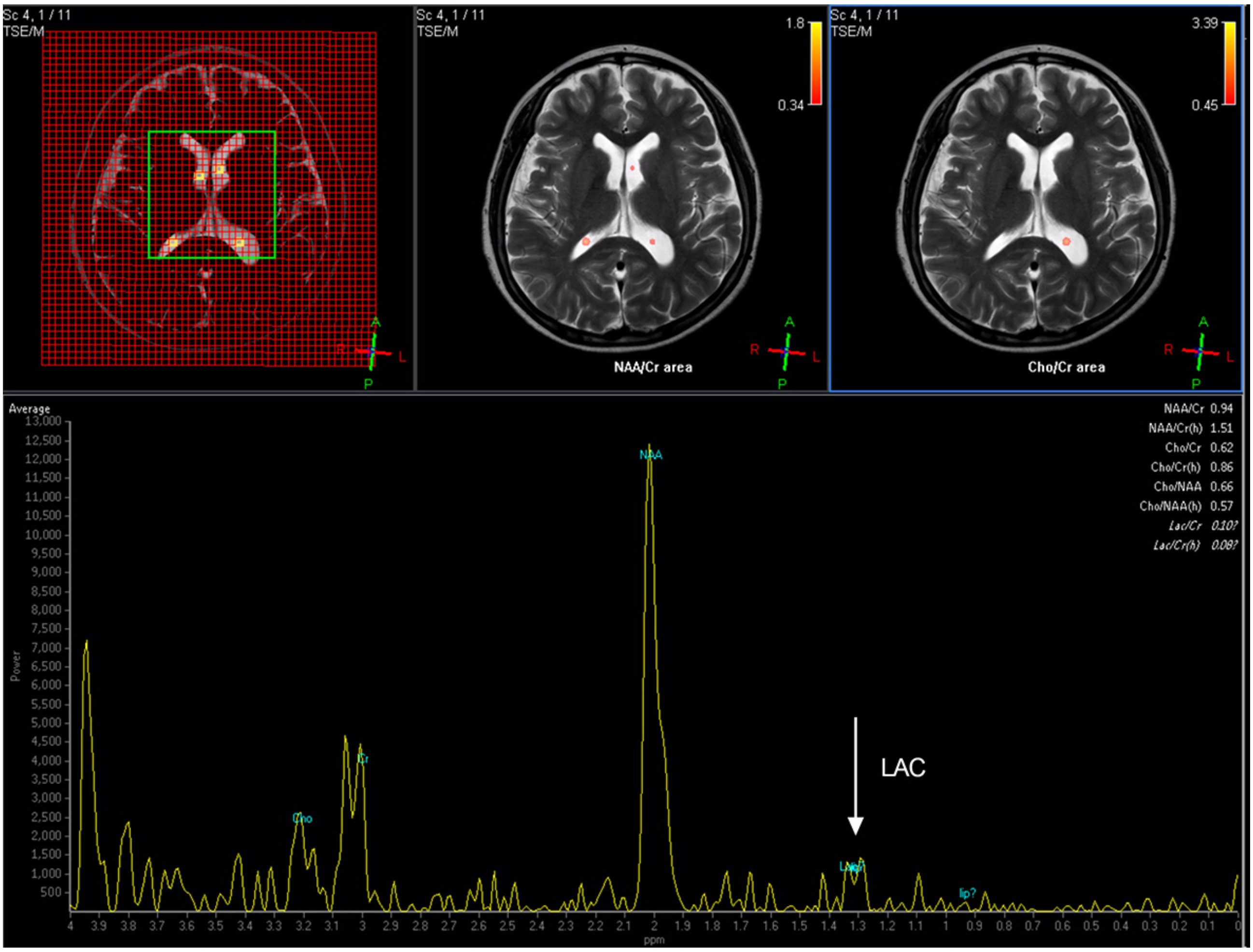

Supplementary references

1. Steele DF, Butler CA, Fox TD. Expression of a recoded nuclear gene inserted into yeast mitochondrial DNA is limited by mRNA-specific translational activation. Proceedings of the National Academy of Sciences of the United States of America 1996; 93(11): 5253-7
2. Rak M, Tetaud E, Godard F, et al. Yeast cells lacking the mitochondrial gene encoding the ATP synthase subunit 6 exhibit a selective loss of complex IV and unusual mitochondrial morphology. The Journal of biological chemistry 2007;282(15):10853-64 doi: 10.1074/jbc.M608692200[published Online First: Epub Date]|.
3. Yu Y, Shi J, Shang S, et al. [Mutations of mitochondrial 12S rRNA gene in type 2 diabetes]. Zhonghua yi xue yi chuan xue za zhi = Zhonghua yixue yichuanxue zazhi = Chinese journal of medical genetics 2001;18(5):388-90
4. Choi SJ, Kim SH, Kang HY, et al. Mutational hotspots in the mitochondrial genome of lung cancer. Biochem Biophys Res Commun 2011; 407(1): 23-7 doi: 10.1016/j.bbrc.2011.02.078 [published Online First: Epub Date]|.
5. Burrage LC, Tang S, Wang J, et al. Mitochondrial myopathy, lactic acidosis, and sideroblastic anemia (MLASA) plus associated with a novel de novo mutation (m.8969G>A) in the mitochondrial encoded ATP6 gene. Molecular genetics and metabolism 2014;113(3):207-12 doi: 10.1016/j.ymgme.2014.06.004[published Online First: Epub Date]|.
6. Clark J, Reddy S, Zheng K, et al. Association of PGC-1alpha polymorphisms with age of onset and risk of Parkinson's disease. BMC Med Genet 2011;12:69 doi: 10.1186/1471-2350-12-69[published Online First: Epub Date]|.
